# Management of Pet Cats: The Impact of the Cat Tracker Citizen Science Project in South Australia

**DOI:** 10.3390/ani8110190

**Published:** 2018-10-24

**Authors:** Philip Roetman, Hayley Tindle, Carla Litchfield

**Affiliations:** 1 School of Natural and Built Environments, University of South Australia, Adelaide, SA 5001, Australia; hayley.tindle@unisa.edu.au; 2 Conservation Psychology and Applied Animal Behaviour Centre, School of Psychology, Social Work and Social Policy, University of South Australia, Adelaide, SA 5001, Australia; carla.litchfield@unisa.edu.au

**Keywords:** citizen science, behaviour change, pet cat management, domestic cats, evaluation

## Abstract

**Simple Summary:**

Domestic cats are popular pets worldwide and play an important role in the lives of many of their owners; however, there is growing awareness of the potential negative impacts of cats. The Cat Tracker citizen science project was conducted in South Australia to better understand domestic cats, their movement, and related community views. The project was deliberately designed to engage cat owners and assist them to make informed decisions about the management of their pet cats. The project collected data through an online social survey and tracking of pet cats, using small GPS units. This study evaluates the project and examines its impact on participant knowledge, attitude, and behaviour. We found that participation in the tracking activity had positive learning outcomes for cat owners and that, after participating, many cat owners placed an increased level of importance on containing cats. Participants reported that they changed their behaviour with existing pet cats and reported intentions to change behaviour with future pet cats. We discuss positive impacts on other members of the community, and how negative impacts may be avoided. We advocate for further research in this area to understand how projects can drive positive changes in knowledge, attitudes, and behaviours.

**Abstract:**

Domestic cats (*Felis catus*) are popular pets worldwide and play an important role in the lives of many of their owners; however, there is growing awareness of the potential negative impacts of cats. Accordingly, there is increasing interest in pet cat management, including changing the attitudes and behaviours of cat owners. The Cat Tracker citizen science project was conducted in South Australia to better understand domestic cats, their movement, and related community views. The project was deliberately designed to engage cat owners and assist them to make informed decisions about the management of their pet cats. The project collected data through an online social survey (*n* = 3192) and GPS tracking of pet cats (*n* = 428), conducted between February 2015 and September 2016. A public report was published in February 2017 and an evaluation survey (*n* = 410) was conducted between March and May 2017. This study evaluates the project and examines its impact on participant knowledge, attitude, and behaviour. We found that participation in the tracking activity had a statistically significant influence on participant-reported learning. For participant cat owners, we recorded statistically significant increases in the level of importance placed on containing cats (both during the day and at night). Participants reported that they changed their behaviour with existing pet cats and reported intentions to change behaviour with future pet cats. We discuss impacts beyond what we set out to measure, including impacts on project onlookers, profound impacts on participants, and how the rebound effect (which can generate negative impacts) may be avoided. We describe social science applied to citizen science and advocate for further research in this area to understand how projects can drive positive changes in knowledge, attitudes, and behaviours.

## 1. Introduction

There is increasing interest in pet cat (*Felis catus*) management, particularly looking to better understand, and sometimes change, the attitudes and behaviours of cat owners [1,2,3,4]. Domestic cats have been companion animals for thousands of years [5]. As popular and cherished pets, domestic cats serve various positive roles in human lives, such as emotional support, love and companionship, and an opportunity for nurturance and empathy [6,7,8]. The interest in cat management stems from the increased awareness of the potential negative impacts of pet cats, such as predation on native wildlife, disease transmission, and annoyance of neighbours [9,10,11,12]. Many owner behaviours such as containing, vaccinating, and de-sexing pet cats may mitigate these impacts of cats [12]. Thus, it is necessary to obtain owner support and involvement in the management of cats. Citizen science projects provide a way to engage cat owners to consider the management of their feline pets.

Citizen science involves public contributions to organised research projects [13]. Volunteer ‘citizen scientists’ can augment the research of professional scientists, most often by collecting or analysing data, but also by contributing to project design, identifying research needs, or reporting results [14]. In addition to the benefits of contributions to research that citizen science projects provide for scientists, there can also be benefits for, and impacts on, the public participants involved. Citizen scientists may find it rewarding, enjoyable, or interesting to contribute to scientific research, and participation may serve as an opportunity to build social relationships, or connections to local places or environments [15,16,17]. Impacts on volunteer participants may also include learning, the development or maintenance of skills, and changing attitudes and behaviours [14,18,19,20]. Participation could also impact more broadly on people and society. For example, through new connections to their environment, people may engage in a variety of conservation behaviours to lead a more sustainable lifestyle [14]. While the potential impacts of participation in citizen science are often discussed in the literature, they are seldom measured.

It is important to evaluate citizen science projects and understand their impacts, although evaluations are often overlooked because of cost and time restraints or the difficulty involved [12,14,21]. The outcomes of project evaluations could help guide the development of future projects by informing reasonable goal setting and describing the range of potential impacts. Additionally, further work is needed to better understand which elements of citizen science projects have an influence on participants. Suggested elements include: hands-on participation, interaction with scientists, and the educational resources that are provided to participants [16,18]. While it is suggested that projects in which participants are involved in more steps of the scientific process may more readily promote learning and changes in behaviour [14], even projects that engage citizens in limited steps (e.g., data collection only) might stimulate change in participants’ knowledge, attitudes, and behaviours. Additionally, further research may lead to improved evaluation methods and models, or assist in the development of funding applications [22].

There are some published studies on the impacts of participation on citizen scientists. For example, participants in the Coastal Observation and Seabird Survey Team (COASST) project learnt how to identify bird species, pursued self-directed learning in other areas of science, and recorded benefits in mental health, confidence, and development of sense of place and place attachment. Furthermore, these participants took additional conservation actions, such as removing rubbish from the beach and telling people of influence about the project findings [23]. Other examples include participants who increased their involvement in butterfly conservation outside of a citizen science project [24], and environmental monitoring research that found outcomes ranging from increasing personal knowledge to changing attitudes, behaviours, and influencing change in natural resource management [19]. Importantly, these authors highlight the mixed results in the field and the need for further evaluations.

We investigated the impact of the Cat Tracker project in South Australia, a citizen science project which sought to further understand domestic cats by investigating cat movement, cat personality, community views on cat management, and owners’ attachment to their pet cats. The project was deliberately designed to engage cat owners and assist them to make informed decisions about the management of their pet cats. While the project made no direct appeal to cat owners to change their behaviour, previous research suggests one of the barriers to cat containment is a lack of understanding of where cats roam, as this information is not typically available to owners [25]. In providing this information to participants we understood that the project may influence cat owner attitudes and behaviours. Similarly, as public engagement was a feature of our approach, we expected the increased awareness and social discussion of cat management issues could also be influential [26]. Our approach was based on previous research suggesting that working with cat owners, rather than focussing on the harmful impacts of their cats and telling them what to do, would be the most effective way to change cat owners’ behaviour [27,28].

## 2. Materials and Methods

### 2.1. The ‘Cat Tracker’ Project Overview

The Cat Tracker project originated in North Carolina, where the protocol for tracking pet cats was developed (see the Acknowledgements section). The Cat Tracker South Australia project followed the same tracking protocols and augmented the American project with the development of detailed social surveys, educational resources for schools, and additional research into cat personality. In South Australia, the project was led by the University of South Australia, with support from local government and a state government board (see the Acknowledgements section).

Participation in the Cat Tracker South Australia project included two key data collection components. First, an online survey examined cat ownership, cat personality, attachment to cats, cat management, and participant demographics. The survey was open to all South Australian residents aged 16+, including both cat owners and non-owners. Non-owners were able to complete the online survey so that analyses of differences in attitudes between cat owners and non-owners could be conducted. Participants were recruited through an open invitation to participate, which ran from February 2015 to September 2016, gaining substantial attention from the media. For example, the project was promoted on commercial television news in South Australia, on a national morning breakfast news television program (*Weekend Today*), on the front page of South Australia’s highest-circulation newspaper (*The Advertiser*), on commercial radio, on public radio (*ABC*) in Adelaide, through regional public radio stations (*ABC*) around South Australia, through community radio stations, and through online news (e.g., *ABC News*). The project was also promoted through social media and community newsletters by the university and government partners.

As part of the online survey, participants were able to select an option to complete a cat personality test for their cat and volunteer to have their cats involved in the second key data collection component of the project: GPS tracking of pet cats. From the cat personality test, participants were sent an individualised report displaying their cat’s personality results (for further information about cat personality and results of the personality research, see Litchfield et al., 2017 [29]). A subset of participants were selected to have their cat tracked using small GPS data loggers [30]. Selection of cats was based on a number of factors related to our research on cat movement, which is not the focus of this paper. Cats were tracked between February 2015 and September 2016. Tracking participants were mailed GPS units with cat harnesses and instructions on how to use this equipment to track their cats. Cat owners removed the GPS units after one week of tracking and mailed the equipment back to the university. The research team then obtained the tracking data from the GPS units and published maps of the cats’ movement on a publically available website.

Results of the project were made available through a detailed public report [30], with a summary available online. The report was published during February 2017. The report included general facts about cats and information about the cats that participated, as well as results of the GPS tracking, including sections on the sizes of the cats’ home ranges, with the differences between ‘sedentary’ and ‘wandering’ cats (determined by the sizes of their home ranges), and statistical relationships between the sizes of cats’ home ranges and the number of roads they crossed, cat fights, and the amount of prey they were seen with. The project’s analyses were based on 3192 survey responses and 428 tracked cats.

### 2.2. Project Evaluation

To enable us to evaluate the Cat Tracker South Australia project, participants who had previously participated in the Cat Tracker project survey were emailed an invitation to complete an online evaluation survey (using SurveyMonkey). Participants who voluntarily chose to complete the survey were provided with information regarding the research and gave informed consent. The evaluation study was approved by the University of South Australia’s Human Research Ethics Committee (#33220; #34155). The evaluation survey comprised of questions relating to participation in the Cat Tracker project. Questions investigated participant learning, cat management, attitudes towards cats, and participation. The evaluation survey was part of a larger online survey examining citizen science participation more broadly. Only the Cat Tracker evaluation component of the survey is discussed here. Relevant questions are provided in Appendix A. Participants completed the evaluation survey between March and May 2017.

Evaluation survey data (*n* = 473) were downloaded from SurveyMonkey. Data were removed if participants did not answer the evaluation component of the larger survey (*n* = 63), leaving a sample size of 410. A further 31 survey responses were of limited value to the evaluation as the respondents only answered one or two evaluation questions. As questions in the evaluation were not compulsory for respondents to answer, sample sizes for each analysis are variable and are provided with the results. Respondents to the evaluation survey could be ‘matched’ to their original Cat Tracker survey responses, allowing some analysis of change of attitudes. Data screening, descriptive statistics, and thematic analyses were conducted in Microsoft Excel, while further statistical tests were conducted in SPSS Statistics 25.3 (IBM Corp., Armonk, New York, USA).

## 3. Results

Respondents to the evaluation survey were all 18 years old or older. The median age cohort was 50–59 years old. The median level of education was a Bachelor degree. There were more respondents who identified as female (*n* = 282) than male (*n* = 64) or Indeterminate/Intersex/Unspecified (*n* = 2).

### 3.1. Learning

We asked participants if they had learnt anything new because of the project, and 71% of participants (*n* = 371) stated that they had. There was some variation among the groups of participants. A higher proportion of cat owners who had participated in tracking reported learning (77%; *n* = 122), compared to cat owners who did not participate in tracking (67%; *n* = 222), and non-owners of cats (74%; *n* = 27). When asked what they had learnt, 239 respondents provided details. For example, “How far the range actually is. It was good to see how they actually cross roads and get into paddocks/etc. How much danger they’re actually exposed to”. These open-ended responses were thematically coded (Table 1).

We conducted a chi-square test for association (*n* = 344) between learning (yes/no) and level of project participation (cat tracked/cat not tracked). There was a statistically significant association between learning and level of participation, χ^2^(1) = 4.069, *p* = 0.044, although the association was only weak to moderate, *φ* = 0.109, *p* = 0.044. The percentage of respondents who reported learning was higher than expected for tracking participants and lower than expected for respondents whose cats were not tracked.

We conducted a chi-square test for association (*n* = 332) between learning (yes/no) and whether respondents had reviewed the results of the project. Respondents were considered to have reviewed the results if they reported they had heard the results (e.g., from a friend or through the media), or if they had read the project results that we provided (e.g., our project report or the online summary of the results). There was a statistically significant association between learning and reviewing the results, χ^2^(1) = 14.350, *p* < 0.001, and the association was moderate, *φ* = 0.208, *p* < 0.001. The percentage of respondents who reported learning was higher than expected for respondents who had reviewed the results and lower than expected for respondents who had not reviewed the results, although this result should be considered tentative, as there were only 19 respondents who had not reviewed the results.

There was also evidence of learning in relation to how far cats travel. We asked participants how far from their house they thought their cat travelled. Results (Table 2) demonstrate that participants who answered the question in both the original project survey (pre-test) and the evaluation survey (post-test) had a better understanding of how far their cat may travel (i.e., there were fewer ‘unsure’ respondents).

To further examine this participant learning, we conducted Wilcoxon signed-rank tests separately on two groups of respondents who had completed the question about how far from home their cat might travel in both the original and evaluation surveys. The first group was the respondents who had participated in cat tracking (*n* = 50). In this group we found a significant increase in the estimated distance their cats travelled from home between the original and evaluation survey responses, *z* = −2.302, *p* = 0.021. The second group was respondents who had not participated in cat tracking (*n* = 73), in which we found no significant difference in responses between the original and evaluation survey responses, *z* = −0.722, *p* = 0.470.

Almost half (46%) of these respondents who had their cats tracked (*n* = 125) stated that their cats went further than they expected. For example, one respondent wrote, “We were staggered that in a cold wintry week [cat name] travelled as far as he did”. The majority (71%) of the tracking participants (*n* = 126) also gave responses when asked if their cat’s tracking results were interesting or surprising. Of this group, 31% mentioned the locations their cats visited, 24% were surprised or interested by the distance cats travelled, 16% commented on the roads or railways that cats crossed, and 13% mentioned the routes that cats had taken. For example, one participant commented, “… crossed a main road on a couple of occasions. It’s very busy and has lots of cars. This was a bit of a shock …”.

### 3.2. Attitude Change

We asked respondents about their attitudes towards containment of pet cats (i.e., keeping cats indoors or contained in an enclosure). Table 3 presents the results for respondents who answered the questions in both the original and evaluation surveys (allowing us to analyse change in attitudes), for both daytime and night-time containment. We conducted paired-samples sign tests to compare the differences between responses in the original and evaluation surveys. There were no significant differences for non-owners of cats, for either daytime or night-time containment. In contrast, there were significant differences for cat owners who had their cats tracked, and for cat owners whose cats were not tracked. The significant differences were found regarding both daytime and night-time containment. The percentage of change is higher for attitudes towards daytime containment. However, night-time containment was more highly supported in the original survey and could not increase to the same extent as attitudes towards daytime containment. The percentage of change (Table 3) reveals greater impact of the project on cat owners who had their cats tracked.

### 3.3. Behaviour Change

We found that 27% of respondents who had their cat tracked (*n* = 119), and 13% of respondents who did not have their cat tracked (*n* = 171) stated that they had changed how they managed their cat. It is important to note that we excluded respondents in the analysis of this question if they nominated their cat as an indoors-only animal (never allowed outside) in the original survey, as they could not change their behaviour to keep their cats indoors more often (the most commonly noted behaviour change in this project).

Respondents then answered a question about how they had changed their behaviour (*n* = 56). The most common change in behaviour was keeping a cat indoors more often (84%), which included bringing it in earlier at night, containing it completely at night, and containing it completely. For example, one participant stated that, “We ensure that he is confined inside at night now and only allow him outside during the day”. Other changes in management related to changing feeding patterns (11%) and provision of items such as a bell, collar and identification (5%). In contrast, two respondents (4%) stated that since the project they were letting their cats outside more often.

To gauge the drivers of these changes, we asked participants why they had changed the way they managed their cats. The main reasons provided (*n* = 56) were to stop their cat hunting and to protect wildlife (45%), to keep the cat safe (for example from dogs, cars, and cat fights; 38%), and because they did not want their cat to roam (23%). For example, “For her safety and the safety of other pets/wildlife”. To investigate future behavioural intentions, we asked respondents if they might manage a new cat differently. Of the respondents (*n* = 253), 50% stated that they would manage a future cat differently, although 7% qualified the statement by stating that it would depend on the cat. Of respondents who would change their management behaviours for a future cat (*n* = 126), 42% stated they would keep their cats indoors more, for example, “I will keep any future cats indoors to protect wildlife”, and another 6% stated that they would not get another cat, for example, “Would not get a cat in future (because of results)”.

### 3.4. Additional Remarks about the Project

We gave participants the opportunity to make additional comments about the project, which were coded into themes (*n* = 222). The most common comments were about participant’s appreciation of the project, enjoyment in participation, or how they found the project interesting (56%). For example, one respondent commented, “Great project! Really enjoyed getting the updates and seeing the data”, and another stated, “It was a great project that was accessible and understandable to ‘non science’ people”. Other themes that emerged were about how the project was educational and raised awareness of relevant issues (16%). For example, one respondent stated that, “I think this was valuable for raising community awareness”, another respondent stated that, “Understanding the cats instead of just ‘fist shaking’ at them is to everyone’s benefit”, and a third respondent stated that, “… I also spoke to our neighbours about trying to keep their cats in at night and they are doing the same thing”. Some respondents stated that they had wished that their cat had been selected for tracking (not all cats volunteered could be tracked; 15%). For example, “I was hoping to have my cat tracked but there must have been a lot of interest in the project!”

## 4. Discussion

The Cat Tracker project was deliberately designed [31] to engage the public and to help them make informed decisions about the management of pet cats. While specific cat management practices were not directly advocated for, the project engaged cat owners with tracking activity and provided cat owners with information relevant to cat management (i.e., maps of the movement of pet cats, information about cat personality, and results of the project that included analyses of both social survey data and cat movement data). We were interested in the impact of the project and recognise that there were four groups of people who could have been influenced:

1. Social survey respondents who tracked their cats (citizen scientists),
2. Social survey respondents who owned cats, but did not participate in tracking (onlookers),
3. Social survey respondents who were not cat owners (onlookers), and
4. Non participants who heard about the project (onlookers).

Members of the four groups may have followed the project and reviewed the results of the project. We consider the participants who were involved in the tracking of their cats to be citizen scientists, as they were involved in the scientific activity that was the key focus of the project (i.e., they tracked their cats). We consider the other three groups to be onlookers [26], as they did not participate in that key activity. However, we do acknowledge that this categorisation is severe, as many people completed the social survey and volunteered to track their cats but were not selected for tracking (i.e., group two). This group certainly participated in the project and volunteered for further participation; however, we were unable to accommodate all volunteers because of time and funding constraints. We have categorised them as onlookers because their realised level of participation was to complete a social survey, which alone would not typically be considered a citizen science project. Future research might find that similar groups of onlookers are more invested in citizen science projects than this categorisation recognises. Indeed, we have found that the differences among the groups are important in understanding the impact and potential impact of the project.

Participants who were most involved in the project (who answered the social survey and had their cats tracked) were most influenced by the project. Participation in the tracking activity had a statistically significant influence on both participant-reported learning and the changes in the distances that cat owners estimated their cats travelled from home (in contrast to participants who did not have their cats tracked). For participant cat owners, we recorded statistically significant increases in the level of importance placed on containing cats (both during the day and at night). While this impact was significant for all participant cat owners, regardless of whether their cat was tracked, the change was greater for those who had had their cats tracked (Table 3). These findings echo literature that suggests the more involved participants are, the greater the impacts of a citizen science project [14,26]. Future research could also examine how demographics might influence impacts on participants. The participant group in our project was mostly females and the median level of education was high; however, we did not have data that made it possible to conduct a robust analysis of the relationships between demographic variables and the impacts of the project.

Our findings demonstrate that specific aspects of project design are particularly important when understanding the impacts of citizen science projects. We found that the level of participation was important, and also that reviewing the results of the project influenced participants (although the sample of participants who had not reviewed the results was small, and thus, these results must be considered tentative—we would welcome more research in this area). The design of the project was also important in enabling us to study its impacts. The protocol included a pre-test (the original survey), an intervention (the tracking activity and reporting of results), and a post-test (the evaluation survey). This sequence may be useful for other citizen science projects, where a survey can be used for participants to volunteer and to gather valuable social data.

It may also be useful to study the long-term impacts of citizen science projects. We found that half of the respondents stated that they would manage their future cats differently because of the project. Most of those respondents stated that they would keep their cats indoors more, and some even stated they would not get another cat. The change in behavioural intentions was reported at a higher rate than the changes in behaviour related to existing pet cats. While some of the increase is likely due to an optimism bias which sees individuals’ overestimate likelihood of future behaviour [32], it is also likely to reflect the difficulties faced and time needed to change the routine and behaviour of existing pets and their own management behaviours. Participants may recognise that with another pet in the future they may be working with a ‘clean slate’ and have the opportunity to more easily shape their pet’s behaviours, expectations, and routines. However, it also needs to be noted that in both cases these behaviours are self-reported and may be influenced by a social desirability bias (the tendency to respond in a way that will be looked upon favourably by others). Further testing of participants would be required to gain a more detailed understanding of the long-term impacts of the project. Additionally, an evaluation immediately after citizen scientists received the results of their tracking might be useful, rather than waiting for the results report to be published. Future evaluations of citizen science projects could study the importance of the timing of various elements of the projects.

While we have measured some impacts of the Cat Tracker project, there are likely to be impacts that we have not measured. We have found some impacts on onlookers who completed our surveys. For example, there were changes in attitudes for cat owners who volunteered for tracking but were not selected to participate (Table 3). Hollow et al. [26] found that non-participant onlookers can learn from, and be influenced by, citizen science projects. We studied two groups of onlookers (survey respondents who did not have their cats tracked and survey respondents who were not cat owners) and found that they both reported learning. As we did not study onlookers who were not involved in the project, there are likely to be additional impacts as many people may have followed the project and been influenced by the activity or the results we provided. We know that the project had reach beyond those who directly participated as media coverage was extensive, with over 50 features in popular media. For example, the Weekend Today television segment had an audience of approximately 280,000 people [33]. We also know that project participants recognised the value of the project in reaching the wider community. For example, participants made largely positive general feedback, mentioning how the project was beneficial in raising awareness about the management of pet cats, and in some cases directly mentioned that they had discussed the project with others. Positive general feedback is encouraging, as previous research has noted that enjoyable experiences in citizen science participation may lead participants to involvement in additional citizen science projects [23]. Future research into onlookers should examine the reasons why some people follow projects but do not actively participate (e.g., our group four), heeding research into the bystander effect [34], and other concepts from social psychology.

We only measured specific impacts, though we recognise that the project had the potential to be influential in other ways. For example, numerous school teachers used the Cat Tracker project in their classrooms. Additionally, there are likely to be participants for whom the project had a profound impact, beyond what was experienced by most participants. We know of one example where a participant engaged numerous neighbours to volunteer their pets for tracking, and organised meetings to discuss the results. The project influenced this participant to keep her cats indoors more, and aroused her curiosity in animal behaviour so much that she reduced her working hours and returned to university to study animal behaviour (this information was shared by the participant during a presentation at the conference of the Australian Citizen Science Association, Adelaide, Australia, 2018).

While we have discussed what we consider to be positive impacts of the project, negative impacts are also possible. Caution needs to be used when highlighting undesired behaviours (e.g., letting cats roam) as although it can bring attention to a problem, it can also illustrate that a behaviour is common and unintentionally convey a social norm [35]. This illustration can lead to a rebound effect, with ‘well-performing’ individuals changing their behaviour (negatively) to be in line with the average [36]. There was some evidence of this occurring, with two respondents stating that after participating in the project they let their cats outside more often. Promoting injunctive norms, which are what is commonly approved and disapproved within the community, may have prevented this small rebound effect [37]. Future projects should continue to highlight the welfare benefits of cat containment and encourage social discussion to demonstrate social norms, and could also utilise injunctive messaging. To negate the rebound effect in an electricity consumption study, Schultz et al. [36] used emoticons (symbols of facial expressions) when providing feedback about household’s energy consumption in comparison to a community average (smiling faces for below-average consumption and sad faces for above-average consumption). During our analysis of cat home ranges [30], we classified the cats we tracked as sedentary (cats with a home range of one hectare or less) or wanderers (cats with a home range of over one hectare). In a similar fashion to Schultz et al. [36], we could have provided positive feedback to cat owners if we found their cat to be sedentary, and negative feedback if their cat was a wanderer. Such an approach is worthy of trials in future projects, particularly where behaviour change is desirable.

While we have demonstrated some impacts of the Cat Tracker project in South Australia, we have also conceived ways in which the impact could potentially have been increased. We reported that most people (both cat owners and non-owners) thought it is important for cats to be inside at night, which may highlight the social norms on this topic. A project that has a more directed objective and message and utilises behaviour change principles (e.g., following a community-based social marketing approach; [38]), or engages participants in a greater degree of participation [31], such as participant-led analysis of the tracking, may result in increased learning and greater changes in attitudes and behaviours. Future projects could engage veterinarians as credible sources of appropriate behaviour [27]. However, care would need to be taken to ensure cat owners are not isolated. Engaging cat owners is essential in projects aiming to improve the management of cats by enabling the community to adopt responsible management practices [3,12].

## 5. Conclusions

Our evaluation has demonstrated that citizen science projects can augment data collection for scientists with impacts on the knowledge, attitudes, and behaviours of project participants and onlookers. The Cat Tracker project was deliberately designed to engage cat owners and enable us to measure the impacts of the engagement. The deliberate design to engage cat owners and assist them to make informed decisions about the management of their pet cats has led to many positive changes in cat-owner knowledge, attitudes and behaviours, without direct appeals for change. The project design, with an online survey, followed by a citizen science activity, followed by an evaluation survey, enabled us to study the impacts of the project. Our results on the changing of attitudes and behaviours are particularly encouraging. Behaviour change is often considered the highest-value potential outcome of public engagement [14,22]. Yet our results are potentially just the tip of the iceberg. There were a substantial but unquantified number of onlookers, who might also have been prompted to change their behaviours. And we uncovered one case where a participant had experienced a profound impact through the project, which might also have been experienced by others. Further work on the design of citizen science projects, with impacts and evaluations in mind, will benefit practitioners of citizen science. In particular, we would like a more sophisticated understanding of various elements of project design, such as the most advantageous formats and timings for the delivery of project results, and methods to evaluate the impacts of citizen science on onlookers and the wider community. The Cat Tracker project and evaluation work has been instructive. Similar projects can be developed to further our understanding of human-animal interactions, including interactions with pets, to improve animal welfare and, we hope, to reduce the impacts of pets on wildlife.

## Figures and Tables

**Table 1:** Thematically coded responses to the question “Please tell us what you learnt as a result of the Cat Tracker project”.

Response Themes	*n*	Percentage
Where cats go and how big their home range is	135	42.9%
Information about their cat specifically	41	13.0%
Community and owner views on cats	29	9.2%
The differences between cats	21	6.7%
Cat personality	17	5.4%
How cats travel: their territories, the methods they take, the paths etc.	15	4.8%
How much cats hunt, keeping cats contained reduces cat hunting	14	4.4%
How many roads cats cross, the danger they are exposed to	9	2.9%
Differences between travel at day and night	7	2.2%
Cats don’t travel as far as we think	6	1.9%
Other (comments made by ≤2 respondents)	21	6.7%

**Table 2:** Responses to the question “How far from your house do you think [YOUR CAT] goes?” (*n* = 261).

Description	Pre-Survey	Post-Survey	% Change
Just on my property	13.0%	17.6%	+35%
100 m beyond my property	29.5%	52.1%	+77%
1 km	5.4%	11.9%	+121%
2 km	0.4%	4.6%	+1100%
Many kilometres	0.8%	3.1%	+300%
Unsure	51.0%	10.7%	−79%

**Table 3:** Changes in attitude towards the containment of pet cats. Percentages for the pre-test and post-surveys are the percentages of respondents who indicated that containment was important or very important on a five-point scale (very unimportant to very important) in response to the statement “Please indicate how important you think it is to contain a cat (e.g., keep the cat inside a house or cat run)?” Sign tests we used to determine if the changes were statistically significant (significant results are marked with an asterisk).

	*n*	Pre-Survey	Post-Survey	% Change	Sign Test Results	Sign Test Differences
**Importance of day-time containment**						
Non-owners	25	64.0%	76.0%	+18.8%	Exact *p* = 0.210	5 Negative 11 Positive 9 Ties
Cat owners, not tracking participants	193	28.5%	60.6%	+112.7% *	*z* = −6.088, *p* < 0.001	24 Negative 90 Positive 79 Ties
Cat owners, tracking participants	114	8.8%	44.7%	+410.0% *	*z* = −4.596, *p* < 0.001	16 Negative 56 Positive 42 Ties
**Importance of night-time containment**						
Non-owners	25	92.0%	96.0%	+4.3%	Exact *p* = 1.000	1 Negative 2 Positive 22 Ties
Cat owners, not tracking participants	209	77.0%	87.6%	+13.7% *	*z* = −4.743, *p* < 0.001	22 Negative 68 Positive 119 Ties
Cat owners, tracking participants	117	65.0%	82.1%	+26.3% *	*z* = −4.491, *p* < 0.001	10 Negative 44 Positive 63 Ties

## Appendix A

Questions from the cat tracker evaluation survey, including only questions which are relevant to the analyses in this paper.

**Demographics** 1. Age: - 18 to 19 - 20 to 29 - 30 to 39 - 40 to 49 - 50 to 59 - 60 to 69 - 70 to 79 - 80 years and above 2. Gender: - Female - Male - Indeterminate/Intersex/Unspecified 3. Highest level of academic achievement: - Year 9 or below - Year 10 or 11 - Year 12 or SACE - Certificate, Diploma, Technical or Trade Qualification - Bachelor Degree - Honours, Graduate Diploma or Masters Degree - Doctoral Degree - Prefer not to answer
**Learning** 4. Because of the *Cat Tracker* project I learnt something new. - Yes - No 5. Please tell us what you learnt as a result of the *Cat Tracker* project: [OPEN-ENDED RESPONSE] 6. How did you participate in Cat Tracker? (select all that apply) - I have read the summary of results online - I have read some of the Cat Tracker report - I have read the entire Cat Tracker report - I read other information about the project - I saw/heard results of the project in the media (e.g., newspaper, radio or television) - Someone told me about the results of the project 7. How far from your house do you now think [INSERT CAT’S NAME HERE] goes? - Just on my property - 100 m beyond my property - 1 km - 2 km - Many kilometres - I’m not sure 8. Did [INSERT CAT’S NAME HERE] travel as far as you expected? - Yes, their travel was what I expected - No, they travelled less than I expected - No, they travelled more than I expected 9. Was there anything that you found surprising or interesting about the places [INSERT CAT’S NAME HERE] went while he/she was tracked? - Yes - No 10. Please describe what you found surprising or interesting about the places [INSERT CAT’S NAME HERE] went while he/she was tracked: [OPEN-ENDED RESPONSE]
**Attitude change** 11. Please indicate how important you think it is to contain a cat (e.g., keep the cat inside a house or cat run)? - Owners should contain cats during the day [RESPONSE OPTIONS: Very important, Somewhat important, Neutral, Somewhat unimportant, Very unimportant] - Owners should contain cats during the night [RESPONSE OPTIONS: Very important, Somewhat important, Neutral, Somewhat unimportant, Very unimportant]
**Behaviour change** 12. Have you changed how you look after or manage [INSERT CAT’S NAME HERE] due to the *Cat Tracker* project? - Yes - No 13. How have you changed how you look after or manage [INSERT CAT’S NAME HERE]? [OPEN-ENDED RESPONSE] 14. Why did you make this change? [OPEN-ENDED RESPONSE] 15. Imagine you get a new cat in the future … Will you look after or manage your new cat differently to the way you manage your current cat/s, due to the *Cat Tracker* project? If so, please explain: [OPEN-ENDED RESPONSE]
**Additional remarks** 16. Do you have any comments about the *Cat Tracker* project?
